# Accumulation of genetic and epigenetic alterations in normal cells and cancer risk

**DOI:** 10.1038/s41698-019-0079-0

**Published:** 2019-03-06

**Authors:** Hideyuki Takeshima, Toshikazu Ushijima

**Affiliations:** Division of Epigenomics, National Cancer Center Research Institute, 5-1-1 Tsukiji, Chuo-ku, 104-0045 Tokyo, Japan

## Abstract

Cancers develop due to the accumulation of genetic and epigenetic alterations. Genetic alterations are induced by aging, mutagenic chemicals, ultraviolet light, and other factors; whereas, epigenetic alterations are mainly by aging and chronic inflammation. The accumulation and patterns of alterations in normal cells reflect our past exposure levels and life history. Most accumulated alterations are considered as passengers, but their accumulation is correlated with cancer drivers. This has been shown for aberrant DNA methylation but has only been speculated for genetic alterations. However, recent technological advancements have enabled measurement of rare point mutations, and studies have shown that their accumulation levels are indeed correlated with cancer risk. When the accumulation levels of aberrant DNA methylation and point mutations are combined, risk prediction becomes even more accurate. When high levels of alterations accumulate, the tissue has a high risk of developing cancer or even multiple cancers and is considered as a “cancerization field”, with or without expansion of physiological patches of clonal cells. In this review, we describe the formation of a cancerization field and how we can apply its detection in precision cancer risk diagnosis.

## Introduction

Human cancers develop due to the accumulation of genetic and epigenetic alterations. Both alterations are now known to be present not only in cancer cells but also in normal cells long before cancer develops. Specific patterns of alterations are associated with exposure to environmental factors. The accumulation is associated with cancer risk and can be utilized for cancer risk diagnosis. Tissue with accumulated alterations is known as a “field for cancerization (cancerization field)”, with or without expansion of physiological patches of clonal cells.

Historically, the measurement of genetic alterations in normal cells has been difficult due to a technical limitation in measuring the extremely low frequency of genetic alterations. However, recent technological advancements have enabled their measurement, and cancer risk assessment using accumulated genetic alterations is now in sight. In this review, we describe the mechanisms by which genetic and epigenetic cancerization fields are induced, its characteristics, and how we can apply the field in precision cancer risk diagnosis.

## Genetic and epigenetic alterations in normal cells reflect an individual’s life history and host responses

### Inducers of genetic alterations

Genetic alterations are induced by aging, mutagenic chemicals, radiation, ultraviolet light (UV), oxygen radical, and other factors (Table 1). Aging is known to be the major cancer risk factor,^1^ and the total number of stem cell divisions, which depends on tissue types, is largely correlated with cancer risk.^2^ Actually, a substantial portion of somatic mutations accumulate early in an individual’s lifetime when stem cells show a high division rate.^1^ In addition to aging, exposure to mutagenic factors induces somatic mutations in human tissues that eventually lead to cancer.^3^ Exposure to UV light^4^ and radiation are also well-known inducers of somatic mutations. Furthermore, oxygen radicals,^5^ constantly produced even in physiological conditions and increased in inflammatory conditions, are also believed to induce somatic mutations.

**Table 1:** Inducers of genetic and epigenetic alterations

Type of alterations	Inducers	Reference
Genetic alterations	Aging	Rozhok et al.^1^
	Mutagenic chemicals	Cogliano et al.^3^
	Acetaldehyde generated from ethanol	
	Aflatoxins from the fungus	
	Benzo(a)pyrene (B[a]P) in incomplete combustion of organic substances, 4-(methylnitrosamino)-1-(3-pyridyl)-1-butanone (NNK) in cigarette smoke, and so on	
	Radiation	
	Ultraviolet light (UV)	Bykov et al.^4^
	Oxygen radicals	Reardon et al.^5^
Epigenetic alterations	Aging	Issa et al.^20^
	Chronic inflammation	
	Ulcerative colitis	Issa et al.^21^
	*H*. *pylori* infection-triggered gastritis	Maekita et al.^22^ Niwa et al.^23^
	HBV or HCV infection-triggered hepatitis	Nishida et al.^24^
	Cigarette smoking	Liu et al.^25^
	Estrogen (in vitro culture)	Cheng et al.^27^

*HBV* hepatitis B virus, *HCV* hepatitis C virus

### Reflections of past exposures by mutation signatures in cancer tissues

**Fig. 1:**
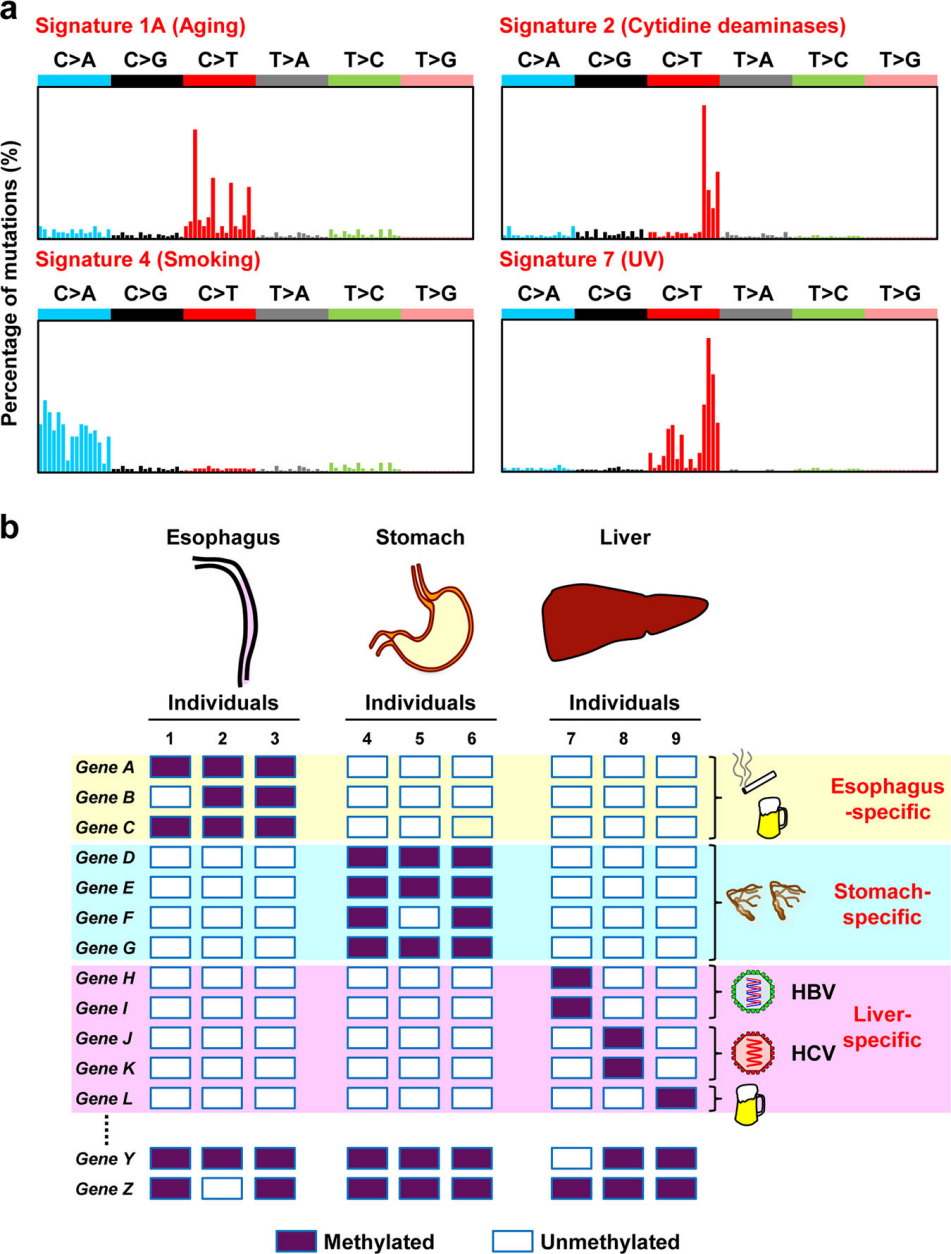
Reflection of past exposure to carcinogens in genetic and epigenetic alterations. **a** Reflection in the mutational signature. Exposure to a carcinogen induces a specific mutation signature (adapted from Alexandrov et al.^7^). **b** Reflection in methylated gene profiles. A different set of genes are aberrantly methylated depending on the tissue types and possibly on the inducers. Unexpressed genes are different among tissues, and specific genes are susceptible to methylation induction in individual tissues, such as *Genes A*, *B*, and *C* in the esophagus, *Genes D*, *E*, *F*, and *G* in the stomach, and *Genes H*, *I*, *J*, *K*, and *L* in the liver. Even among liver-specific susceptible genes, genes are methylated reflecting the cause of inflammation, such as *Genes H* and *I* by HBV infection, *Genes J* and *K* by HCV infection, and *Gene L* by alcohol

Exposure to a specific agent induces a specific combination of mutations, referred to as a “mutation signature” (Fig. 1a).^6,7^ Such mutation signatures have been mainly identified in cancer tissues, in which both driver and passenger mutations can be readily detected due to clonal expansion of cancer cells. Most recently, approximately 85,000,000 mutations in more than 23,829 cancers were classified into single base substitutions (SBS) in 96 trinucleotide contexts, doublet base substitutions (DBS), and small insertion and deletions (ID).^6,7^ Non-biased analysis was able to extract 67 characteristic patterns of SBS, and 49 of them were considered to be associated with exposure to specific carcinogens. Signature 1, characterized by C>T transitions at NpCpG trinucleotides, is associated with aging; signature 7, reflecting a large number of CC>TT dinucleotide mutations at dipyrimidines, with UV light; signature 4, characterized by C>A transversions with strand bias, with cigarette smoking; signature 2, characterized by C>T and C>G mutations at TpCpN trinucleotides, with excessive activity of cytidine deaminases (AID/APOBEC), which is observed in chronic inflammation. Mutation signatures in cancer tissues are composed of mutations accumulated not only before cancer development but also after development.

Specific patterns of copy number alterations can be also present in cancer tissues, referred to as a “copy number signature”. For example, copy number signatures in high-grade serous ovarian cancers can predict overall patient survival and the probability of platinum-resistant relapse.^8^ However, it is still unclear whether or not the copy number signature reflects past exposure to specific carcinogens.

### Mutation signatures in normal tissues

Since a signature of somatic mutations in normal tissues is least influenced by the biological selection of specific mutations, it can provide vital information about past exposure to specific carcinogens. At the same time, a normal tissue is composed of a large number of clonal patches of cells,^9^ and a specific mutation, if ever, is present only in one of the many patches. DNA with the mutation constitutes only a very minor fraction in DNA from the bulk tissue, and it used to be very difficult to detect such somatic mutations.^10^ Thankfully, the development of new cutting-edge detection technologies^11–14^ has now enabled detection of rare mutations in a normal tissue exposed to a specific carcinogen,^15^ and mutation signatures can be now analyzed in normal tissues.

Signature 7 was detected in normal skin tissues, which are exposed to UV light.^16^ C>T transitions at GpCpN trinucleotides were detected in normal gastric mucosae exposed to gastritis triggered by *Helicobacter pylori* (*H*. *pylori*) infection, which induces up-regulation of AID.^17^ Signature 1 and signature 5, T>C transitions at ApTpN trinucleotides, were predominant in normal esophageal tissues,^18,19^ and C>A transversions were frequently detected in those of individuals with a severe smoking history.^15^ The signatures in these normal tissues were in good accordance with those in cancer tissues due to the same carcinogens. Therefore, mutation signatures reflect past exposure to carcinogens, namely an individual’s life history.

### Inducers of epigenetic alterations

Epigenetic alterations also appear to be induced by exposure to various environmental stimuli (Table 1). Aging was first shown to be correlated with increased levels of aberrant DNA methylation, possibly as a function of somatic cell replications.^20^ Subsequently, chronic inflammation triggered by various factors was also shown to be causally involved in inducing aberrant DNA methylation.^21–24^ Additionally, cigarette smoking was reported to induce aberrant DNA methylation in vitro,^25^ which was in line with the in vivo finding of high DNA methylation levels in normal esophageal tissues of individuals with a long smoking history.^26^ Estrogen treatment was also reported as an inducer of aberrant DNA methylation in cultured mammary epithelial cells.^27^

### Reflection of past exposure and host responses in methylated gene profiles

A different set of genes are methylated depending on the tissue types (tumor types)^28^ and are likely methylated depending on the inducers (Fig. 1b). Mechanistically, genes that undergo DNA methylation are instructed by pre-existing epigenetic modifications.^29^ Namely, genes with high levels of trimethylation of histone H3 lysine 27 (H3K27me3) are susceptible to induction of aberrant DNA methylation,^30–32^ and genes lacking RNA polymerase II (pol II) binding are also likely to become methylated.^33^ Because the H3K27me3 status and distribution of pol II are different among the tissue types and are altered by exposure to different inducers, such as oxidative damage and colitis,^34,35^ exposure to a specific inducer likely leads to methylation of a specific set of genes according to the tissue type. Chemicals, such as cobalt compounds and cigarette smoke condensate, were also reported to induce alterations of the H3K27me3 status.^25,36^

Phenotypically, carcinogen-specific induction of aberrant DNA methylation has been demonstrated in hepatocellular carcinomas (HCCs) and cirrhotic liver tissues associated with HBV infection, HCV infection, or alcohol.^37,38^ HCV infection induced extensive methylation at more than 18,000 unique CpG sites, while HBV infection and alcohol-induced moderate methylation at 400–600 unique CpG sites, respectively.^37^ Once aberrant DNA methylation is induced at specific genes, the methylation profiles can persist throughout an individual’s lifetime, even after the inducer is no longer present.^39^

The accumulation level of epigenetic alterations reflects not only the type of inducer but also the duration of exposure to the inducer.^39^ Additionally, the accumulation level can reflect the differences in host responses to an environmental stimulus, which are determined by a single nucleotide polymorphism (SNP) of a specific gene. A well-established example is the influence of the *IL1B* genotype, which influences gastric cancer incidence in *H. pylori*-infected populations,^40,41^ on DNA methylation levels in normal gastric tissues.^42^ Individuals who have SNPs that secrete more IL-1β in response to *H. pylori*-triggered inflammation have higher levels of aberrant DNA methylation and higher risks of gastric cancer.

Taken together, analyzing genetic and epigenetic alterations accumulated in normal cells can provide information on an individual’s life-time exposure to environmental factors that induce genetic and epigenetic alterations that may eventually lead to cancer.

## Formation of a field for cancerization by genetic and epigenetic alterations

### What is the field for cancerization?

**Fig. 2:**
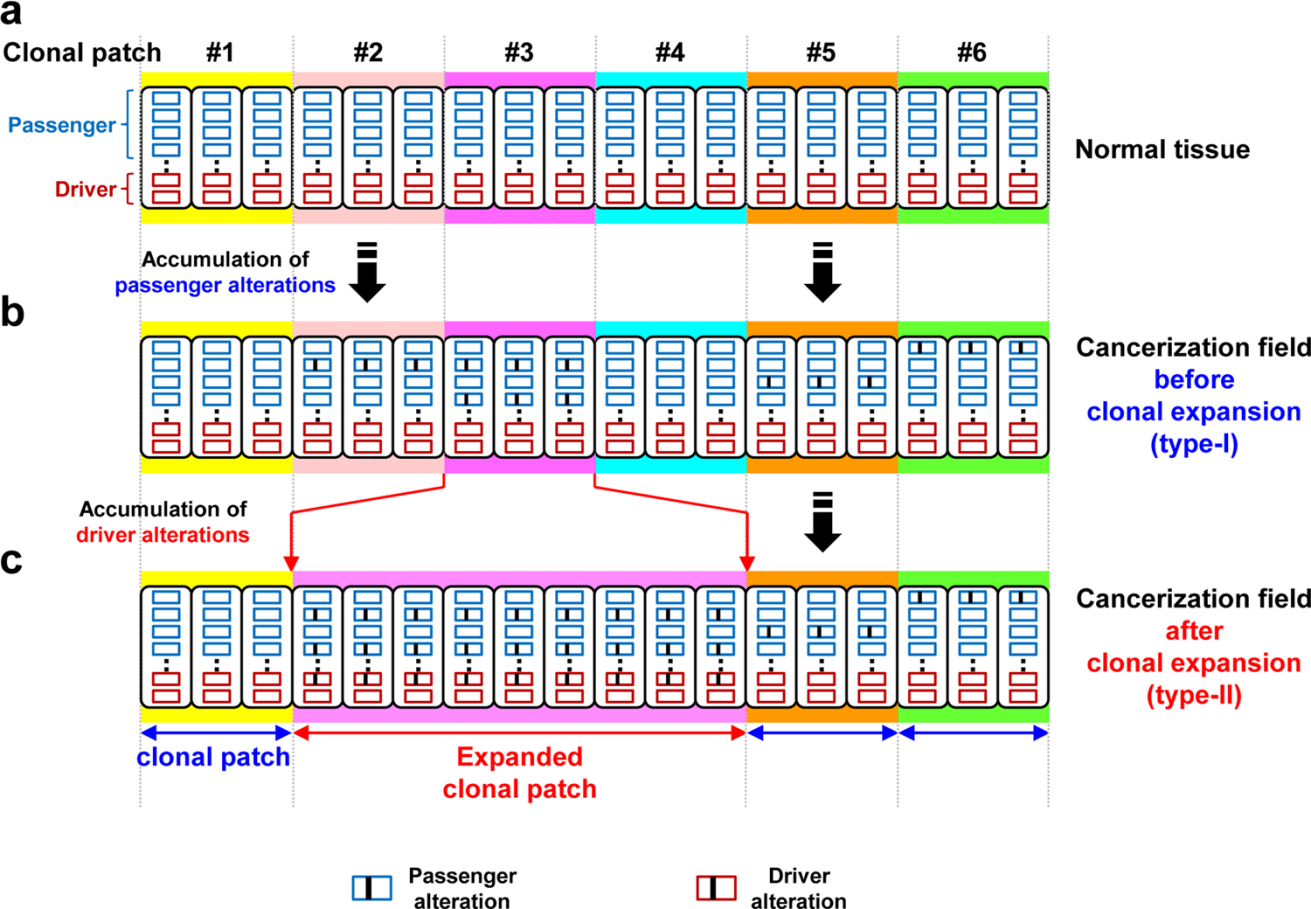
Formation of a field for cancerization. **a** Normal tissues are assembled from clonal patches of normal cells. **b** Genetic and epigenetic alterations are potently induced by exposure to specific inducers, and passenger alterations are mainly accumulated in normal tissues without expansion. **c** Both passenger and driver alterations, which can induce monoclonal cell proliferation, are accumulated in normal tissues with expansion

Some cancers, particularly those associated with chronic inflammation, often develop at multiple foci in a tissue. This phenomenon is known as the concept of “field cancerization” or “a field for cancerization”.^43–45^ The concept was first proposed by Slaughter et al. based upon the presence of multiple microscopic cancers in grossly normal mucosa of patients with oral squamous carcinomas.^43^ The finding was further advanced by detecting *TP53* mutations in clonally expanded patches of cells, which may or may not be detected by routine microscopic examination, in multiple types of cancers (Fig. 2c).

In contrast, even without the expansion of clonal patches of cells, the cancerization field can be formed by accumulating mutations and/or aberrant DNA methylation. Especially, passenger alterations are far more frequent than driver alterations,^15,46,47^ and can be already accumulated in normal tissues without expansion of clonal patches (field type-I, Fig. 2b). The accumulation of passenger alterations is likely to be associated with the accumulation of driver alterations that have not yet caused the expansion of clonal patches. In contrast, driver alterations will eventually cause the expansion of clonal patches, and thus both passenger and driver alterations are accumulated in normal tissues with the expansion of clonal patches (field type-II, Fig. 2c). Such expansion of clonal patches is considered to be histologically normal initially, but can eventually lead to the formation of histologically distinguishable premalignant lesions.

Experimental demonstration of the presence of a cancerization field, however, has been difficult again because of the extremely low frequency of genetic alterations accumulated in normal cells. Historically, to overcome this issue, multiple transgenic animal models with marker genes were developed.^48^ A mutation in a marker gene can be selected by observing the altered function of the gene, such as plaque color and drug resistance, and rare mutations can be measured.^49,50^ Using these animal models, the frequency of somatic mutations in normal tissues was found to be in the levels of 10^−6^ to 10^−4^ per gene even after exposure to high levels of mutagenic chemicals. The animal models also showed increased mutation frequencies by exposure to various carcinogens.

### Detection strategies for rare mutations in normal tissues

In human tissues, as repeatedly mentioned, the detection of rare mutations was previously very difficult. However, it was recently achieved by (1) using samples containing a limited number of clonal patches,^16,18,19,51^ and (2) enhancing the accuracy of next-generation sequencing.

The sampling strategy of only a limited number of clonal patches from a small piece of tissue was used to measure mutations accumulated in histologically normal human skin tissues^16^ and esophageal tissues.^18,19^ Epithelial organoids expanded from a single stem cell were used to measure mutations accumulated in normal adult tissue stem cells of the small intestine, colon, and liver.^51^ These studies revealed rare mutation characteristics of ultraviolet exposure or tissues of the origin. However, because of the high sequencing cost of analyzing a large number of human clinical samples, the association of these mutations with cancer risk could not be robustly established.

To measure rare mutations using next-generation sequencing, it is important to distinguish true mutations in a single or small number of DNA molecules from sequencing errors that arise in up to 1% of sequence reads covering a specific base position due to errors during PCR amplification and sequencing. This issue was overcome by multiple strategies for the preparation of sequencing libraries. By tagging individual DNA molecules with unique molecular barcodes, a true mutation is detected as a mutation in multiple sequence reads with the same barcode.^10^ Some of the molecular barcode-based methods, such as duplex sequencing,^12^ can distinguish a true mutation on both strands of a DNA duplex (fixed mutations) and a variation on either strand of a DNA duplex (derived from a pre-mutagenic lesion or a PCR error at early PCR cycles). However, these methods require a large number of sequencing reads, and it is very costly to analyze a large number of samples because of high sequencing cost.

To reduce sequencing cost, methods using a small number of template DNA molecules have been developed. Bottleneck sequencing improved the molecular barcoding system by adding a simple dilution step before amplification of the sequencing library.^13^ We also developed the 100 copy-based method, in which accurately quantified 100 copies of genomic DNA are used as a PCR template for amplicon sequencing and a true mutation (variant allele frequency, 1%) can be distinguished from sequencing errors (frequency, less than 1%).^14^ However, these methods cannot detect mutations at specific base positions, such as activating mutations of oncogenes. Bottleneck sequencing, which also adapts molecular barcodes, can distinguish a true mutation and a pre-mutagenic lesion.

Furthermore, the enhancement of the accuracy of next-generation sequencing has been also achieved by developing computational and statistical strategies to exclude sequences of low confidence, such as sequencing errors.^16,18,52^ Sequencing errors are excluded by comparing the frequencies of detected mutations with those of the background sequencing errors. In addition, the detection methods of somatic mutations at a single-cell level are now being attempted.^53^ However, current methods suffer from amplification bias and the high cost of library preparation, and multiple methods to reduce the bias and cost are being developed.^52,54^

### Involvement of genetic alterations in a field

**Fig. 3:**
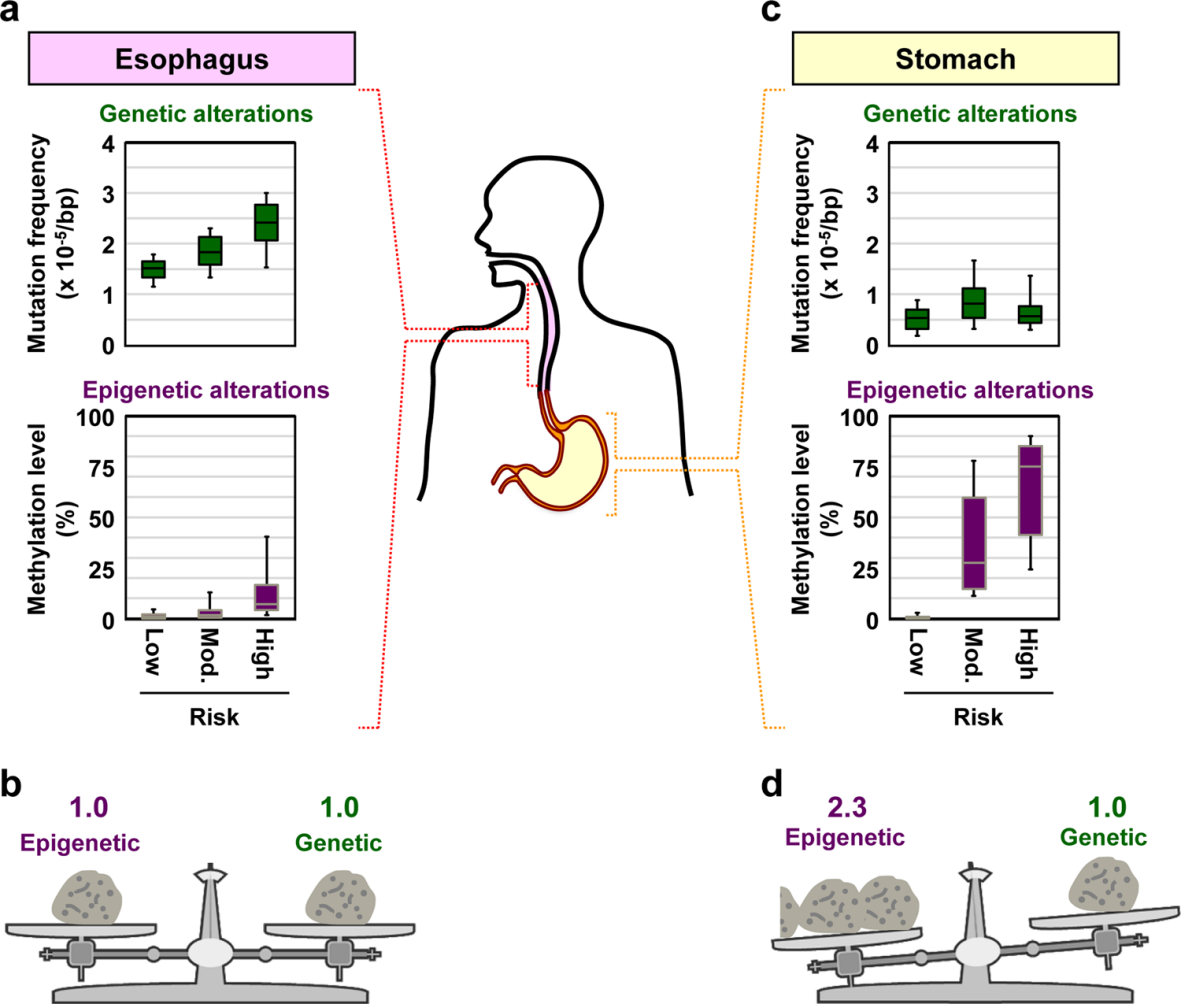
Different impacts of genetic and epigenetic alterations on cancer risk. Both genetic and epigenetic alterations are involved in forming a field for cancerization, but their relative contributions differ depending on the cancer types. **a**, **b** The impacts of genetic and epigenetic alterations are similar for ESCCs. Box plots represent median (center line), upper, and lower quartiles (box limits), and 91 and 9 percentiles (whiskers). The original data were obtained from our previous publication.^15^ **c**, **d** The impact of epigenetic alterations is higher than that of genetic alterations for gastric cancer

The impact of accumulation of point mutations in normal tissues on cancer risk was demonstrated using the 100 copy-based method. Frequencies of point mutations were measured in esophageal tissues with a low, intermediate, or high risk of esophageal squamous cell carcinoma (ESCC) (Fig. 3a). The mutation frequency in esophageal tissues (non-cancerous tissues) of ESCC patients (high-risk group) was 1.4-fold higher than that in esophageal tissues of healthy people without any exposure to ESCC risk factors (low-risk group). Even among people exposed to the risk factors, the mutation frequency in the high-risk group was higher than that in the intermediate-risk group (healthy people with exposure to the risk factors). The 100 copies of genomic DNA were obtained from a piece of biopsy (about 0.75 mm^3^), corresponding to about 750,000 cells, and were likely to contain a large number of patches. Therefore, the mutations detected were likely to be derived from independent patches, and to be involved in cancerization field type-I. In contrast, in normal gastric tissues, a risk level-dependent increase in the mutation frequency was not observed (Fig. 3c). Namely, the mutation frequency in non-cancerous gastric tissues of gastric cancer patients (high-risk group) was similar to that in healthy people with *H*. *pylori* infection.

Most accumulated genetic alterations are considered as passengers, but their accumulation is correlated with cancer drivers. The functions of mutations accumulated in normal tissues can be assessed by the ratio of non-synonymous mutations/synonymous mutations. In normal skin tissues, *NOTCH1* and *NOTCH2* mutations showed a significant excess of non-synonymous mutations, and clustered in the extracellular epidermal growth factor-like domain.^16^ In normal esophageal tissues, *NOTCH1* mutations were present in 25–42% of the cells, and were considered to be associated with clonal expansion.^18,19^ In benign lesions of melanoma patients, which are clearly with clonal expansion, BRAF V600E mutation was detected.^55^ In normal blood cells, specific chromosomal alterations were detected in expanded clonal cells, and were associated with increased risk of hematological malignancies.^56^ These mutations are considered to be involved in the formation of cancerization field type-II, which is associated with the expansion of clonal patches.

Experimentally, the possibility of contamination of an extremely small number of cancer cells in non-cancerous tissues cannot be completely excluded, as HER2-positive cancer cells were detected in adjacent normal breast tissues.^57^

### Involvement of epigenetic alterations in a field

The impact of accumulation of aberrant DNA methylation on cancer risk was shown much more easily because its accumulation levels in normal tissues are high and can be readily measured. In the stomach, DNA methylation levels in non-cancerous gastric tissues increased in the order of healthy people without *H. pylori* infection (low risk), healthy people with past *H. pylori* infection (intermediate risk), patients with a single gastric cancer, and patients with multiple gastric cancers (high risk).^22,58^ In the mammary glands, the DNA methylation levels of *RASSF1A* in non-cancerous tissues of breast cancer patients were higher than those in reduction mammoplasty (normal) tissues.^59^ Additionally, the presence of an epigenetic field for cancerization was shown for colon cancers,^60^ esophageal adenocarcinomas,^61,62^ liver cancers,^24,63^ urothelial cancers,^64^ and cervical cancers.^65^

Most epigenetic alterations accumulated in normal tissues are considered to be passengers, which are involved in cancerization field type-I. However, their accumulation levels are correlated with those of drivers of cancer. Passenger genes tend to be methylated in a larger fraction of normal cells compared to driver genes. Therefore, measuring DNA methylation levels of passenger genes is technically easy and thus accurate, and is considered to be suitable for cancer risk diagnosis. As for the epigenetic alterations of driver genes, methylation-silencing of several tumor-suppressor genes, such as *CDKN2A* (*p16*), *CDH1* (E-cadherin), and *SMARCA1*^66^ has been reported. Alterations in these driver genes accumulate only at low levels but are considered to be causally involved in the formation of a field. Taken all together, accumulation of genetic and epigenetic alterations in normal tissues can be associated with cancer risk.

## Different impacts of genetic and epigenetic alterations and their combination

Both genetic and epigenetic alterations are involved in forming a field for cancerization, but their contributions differ depending on the cancer type.^15^ In normal esophageal tissues, both the mutation frequency and DNA methylation levels increased according to the risk level of ESCCs (Fig. 3a), indicating that the impact of genetic and epigenetic alterations on cancer risk is similar for ESCCs (Fig. 3b). In normal gastric tissues, DNA methylation levels increased in a risk level-dependent manner, but the mutation frequency did not (Fig. 3c). The impact of epigenetic alterations was 2.3-fold higher than that of genetic alterations in the gastric tissues (Fig. 3d).

The different impacts of genetic and epigenetic alterations on cancer risk are considered to depend on the major carcinogens involved and their carcinogenic mechanisms. In the esophagus, cigarette smoking and alcohol intake, which induce both genetic and epigenetic alterations, are major risk factors of ESCCs. Therefore, the impact of genetic and epigenetic alterations is considered to be similar in the esophagus. In the stomach, chronic inflammation triggered by *H*. *pylori* infection, a strong inducer of aberrant DNA methylation, is nearly an exclusive risk factor for gastric cancers. Therefore, the impact of epigenetic alterations is considered stronger in the stomach.

The involvement of both genetic and epigenetic alterations in ESCC risk led to the idea that combining both alterations can achieve a more precise risk diagnosis.^15^ Traditional risk factors only (aging, cigarette smoking, alcohol drinking, and betel quid chewing) showed a reasonable predictive power (c-index; range 0–1) of 0.851. The addition of the mutation frequency to the traditional risk factors improved the power to 0.894, and further addition of the methylation level improved the prediction power even more to 0.926. This showed that the combined measurement of both genetic and epigenetic alterations can achieve precise cancer risk estimation, and provide cancer risk information that cannot be obtained from life history of exposure to traditional risk factors. Additionally, how genetic and epigenetic alterations are combined should be optimized depending on the cancer types due to their different impacts.

## Clinical implementation of precision cancer risk diagnosis

**Fig. 4:**
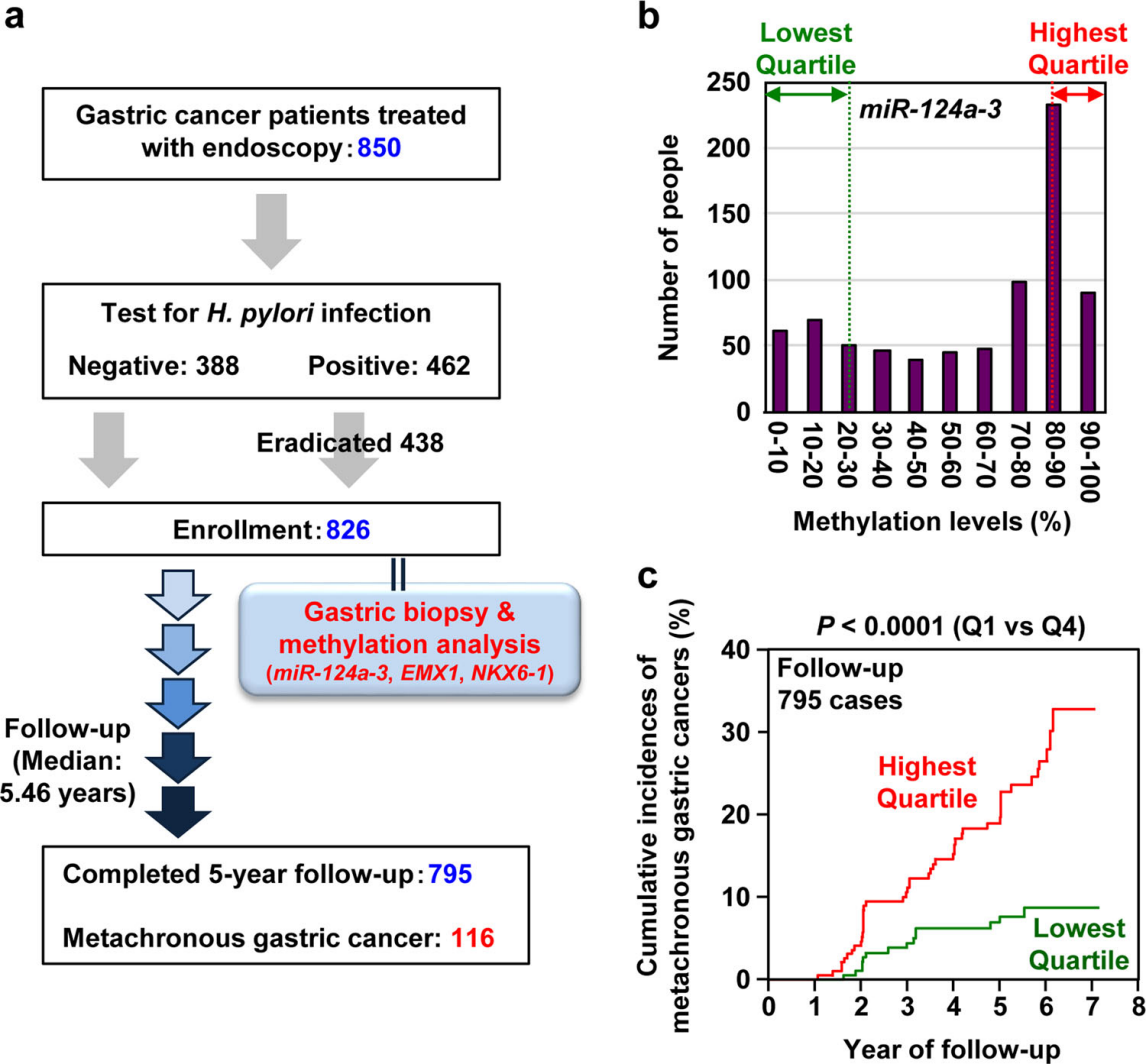
A multicenter prospective clinical study to predict cancer risk by measuring accumulated alterations. **a** Study design of the prospective study. Among the 826 patients enrolled, 116 patients developed metachronous gastric cancer after 1 year of enrollment with a median follow-up period of 5.46 years. **b** Distribution of the DNA methylation level of one of the pre-selected marker genes, *miR-124a-3*, in non-cancerous gastric tissues. Patients in the highest quartile had DNA methylation levels of 88.1–91.8%, while those in the lowest quartile had those of 8.3–23.0%. **c** The impact of methylation burden on the risk of metachronous gastric cancer. The highest quartile had a 3-fold higher risk of developing a metachronous gastric cancer than the lowest quartile. Risk prediction among patients who have already been treated for the first cancer is generally very difficult, but was achieved by measuring the methylation burden. This figure was modified from our previous study^68^

Even a prospective clinical study has demonstrated the usefulness of measuring the accumulation of alterations in normal tissues for cancer risk diagnosis. It has been considered and is now shown that aberrant DNA methylation (methylation burden) has a much larger impact on gastric cancer risk than mutations. Therefore, in this prospective study,^67,68^ only the methylation burden in a gastric mucosa was measured for 826 gastric cancer patients treated by endoscopic submucosal dissection (Fig. 4a). Only three pre-selected marker genes were measured to avoid multiple testing. After a median follow-up period of 5.46 years, patients who had the highest methylation burden (highest quartile) had a 3-fold higher risk of developing a metachronous gastric cancer than those who had the lowest burden (Fig. 4b, c).

## Perspective

Determining an individual’s future cancer risk by measuring genetic and epigenetic alterations is a promising approach for precision cancer risk diagnosis. One potential limitation is that the target tissue needs to be collected for this strategy. However, the recent clinical practice involves routine biopsies of a variety of organs, such as gastrointestinal tract, liver, skin, prostate, and breast. Additionally, the causal involvement of epigenetic alterations is now suggested for various human chronic disorders, such as neurodegenerative and metabolic disorders. Therefore, risk diagnosis by measuring epigenetic alterations has the potential to be expanded to various human disorders other than cancer.
